# Prediction of cardiac arrest in patients with heart failure in Sweden: a registry study with development of a machine learning model

**DOI:** 10.1136/bmjopen-2025-113890

**Published:** 2026-06-25

**Authors:** Meena Thuccani, Araz Rawshani, Johan Herlitz, Christian Rylander, Peter Lundgren

**Affiliations:** 1 Department of Molecular and Clinical Medicine, University of Gothenburg, Gothenburg, Sweden; 2 Department of Cardiology, Sahlgrenska University Hospital, Gothenburg, Sweden; 3 Prehospen—Centre for Prehospital Research, University of Borås, Borås, Sweden; 4 Anaesthesiology and Intensive Care, Uppsala University, Uppsala, Sweden; 5 Center for Digital Health, Sahlgrenska University Hospital, Gothenburg, Sweden

**Keywords:** Heart failure, Machine Learning, Prognosis, Cardiopulmonary Resuscitation, Death, Sudden, Cardiac, Out-of-Hospital Cardiac Arrest

## Abstract

**Abstract:**

**Objective:**

30-day survival after cardiac arrest is low, 12.4% and 36% for out-of-hospital and in-hospital cardiac arrest, respectively. Heart failure is a known risk condition for cardiac arrest. Improving our ability to identify patients at high risk of cardiac arrest would enable prevention. We aimed to develop a prediction model for cardiac arrest to be used in patients newly diagnosed with heart failure.

**Design:**

A nationwide registry-based observational study.

**Setting:**

Data were sourced from the Swedish Heart Failure Registry (1 January 2005 to 31 December 2021).

**Participants:**

This cohort included 45 068 patients discharged from hospital after first hospitalisation for newly diagnosed heart failure. Patients discharged from hospital with palliative care and/or implantable defibrillators were excluded.

**Outcome measure and analysis:**

The primary outcome was defined as cardiac arrest registered in the Swedish Registry for Cardiopulmonary Resuscitation until final follow-up (15 November 2022). Patients who died without resuscitation were treated as competing events. A Random Survival Forest model for competing risk was developed using predictors from the heart failure registry. The model was evaluated with Brier score, observed versus predicted cumulative incidence, Concordance-index (C-index) and time-dependent area under the curve of a receiver operating characteristics graph (AUC-ROC).

**Results:**

In this cohort, 2399 (5%) patients had received cardiopulmonary resuscitation (CPR) (5%), and 31 989 (71%) patients died without resuscitation. Our model with 82 predictors had a low Brier score indicating a capacity to accurately predict cumulative incidence of cardiac arrest on a group level. However, the model also had a low C-index 0.52 and low AUC-ROC 0.63–0.65.

**Conclusion:**

Our Random Survival Forest model for competing risk could not accurately predict cardiac arrest in individual patients newly diagnosed with heart failure, because the event death without attempted resuscitation was treated as a competing event. The lack of information on transitions to palliative care and Do-Not-Attempt-CPR-orders limits the clinical relevance of any cardiac arrest prediction model.

**STRENGTHS AND LIMITATIONS OF THIS STUDY**

- In this study we used Random Survival Forest for competing risk, which is a robust machine learning algorithm that can perform survival analysis with competing risk on high dimensional data.
- Model performance might improve by higher number of decision trees and more repeats in the cross-validation in the training process.
- This model does not reflect changes in patients’ health over time. A shorter observation time might improve prediction accuracy.
- Model was trained with a large dataset with patient data obtained from the Swedish Heart Failure Registry, which due to limited completeness is not representative of the entire Swedish heart failure patient population.

## Background

 Although survival after cardiac arrest has improved in Sweden over the last two decades, 30-day survival still remains low at 12.4% and 36% for out-of-hospital cardiac arrest (OHCA) and in-hospital cardiac arrest (IHCA), respectively.^1^ Similar survival rates, if not lower, are reported globally.^2 3^ Even if resuscitation and post-resuscitation care will improve in the future, cardiac arrest will still carry a major risk of death from cardiac mechanisms or hypoxic/ischaemic brain injury. Avoiding cardiac arrest by preventive measures in people at risk would be more effective to decrease fatalities and poor outcomes. Methods to predict cardiac arrest would enable preventive measures for individuals at risk.^4^

Heart failure is associated with sudden cardiac death (SCD) and cardiac arrest,^5 6^ and is thought to be a risk condition for cardiac arrest. One prophylactic intervention used for a subset of these patients is the implantable cardioverter defibrillator (ICD). This improves the overall survival and prevents decline in quality of life in this patient group.^7 8^ Pharmacological treatment with ACE inhibitors, aldosterone receptor antagonists and beta-blockers has shown to reduce mortality among patients with heart failure.^9^ With the current strategy of prevention, mortality among these patients has declined yet still remains high at 58% 5 years after first diagnosis.^10^

Risk stratification of mortality in patients with heart failure is used to allocate appropriate primary prevention against condition deterioration and mortality. Previous findings have indicated that non-sustained ventricular tachycardia (VT) on Holter monitoring and abnormalities on signal average ECG combined with left ventricular ejection fraction (LVEF) <30% and coronary artery disease are predictors of high risk for arrhythmias and SCD. A positive electrophysiologic test with inducible sustained VT in patients with coronary artery disease was predictive of SCD. However, a negative result did not exclude the risk of SCD. Meng *et al* aim to develop prediction models to predict SCD in patients with heart failure and LVEF <35%.^11^ Researchers from Japan were able to develop an artificial intelligence model based on ECG data combined with New York Heart Association (NYHA) class and LVEF to predict SCD in patients with heart failure. This model had a better performance than using conventional risk factors alone.^12^

In this study we aim to develop a machine learning prediction model to be used on hospital discharge for patients newly diagnosed and treated for heart failure. This model may predict cardiac arrest after hospital discharge and thus identify the patients in need of further preventive interventions.

## Method

### Study design and population

This retrospective observational study included adult (≥18 years) patients with a new diagnosis of heart failure identified in the Swedish Heart Failure Registry (SwedeHF). These were patients who had been discharged alive from hospital after their first hospitalisation for heart failure from 1 January 2005 to to 31 December 2021. Patients with palliative care at hospital discharge or ICD/cardiac resynchronisation therapy with defibrillator (CRT-D) at hospital discharge were excluded. Patients discharged from hospital with palliative care would not be eligible for preventive measures against cardiac arrest contradictory to their palliative treatment strategy. We have therefore excluded them from our study population which our model was based on. Patients discharged with ICD/CRT-D are patients with a high risk of developing a ventricular arrhythmia. These patients therefore had a prophylactic device against cardiac arrest. This is why we have excluded these patients as well. The data from the heart failure registry were linked with the Swedish Registry for Cardiopulmonary Resuscitation (SRCR), using their unique personal identification number, and merged into a pseudonymised dataset. No sample size calculations were made as the sample size was deemed sufficient.

This study is reported in accordance with the Strengthening the Reporting of Observational Studies in Epidemiology guidelines and Transparent Reporting of a multivariable prediction model for Individual Prognosis or Diagnosis+Artificial Intelligence statement.^13 14^ This study was not registered, and a study protocol was not published.

### Swedish Heart Failure Registry

The SwedeHF is a national quality registry started in 2003 with the aim to improve the treatment and care for heart failure patients in Sweden. Currently, most registrations include the first registration after a hospital admission for newly diagnosed heart failure. In 2020 the registry’s quality control found that only 13% of patients newly diagnosed with heart failure were reported to the registry nationwide, with varying reporting rates between hospitals.^15^

### Swedish Registry for Cardiopulmonary Resuscitation

The SRCR is a national quality registry for cardiac arrests where cardiopulmonary resuscitation (CPR) was initiated. This registry includes both OHCA and IHCA. The data are registered in accordance with the Utstein framework by respective ambulance personnel and nurse/doctor responsible for the patients care.^16^ Cross-checking of the IHCA registry within SRCR against medical records has demonstrated a registry completeness of 77%.^17^ Similarly, the OHCA registry has a 75% completeness.^18^ However, retrospective reporting has improved the completeness in recent years with regard to OHCA.

### Study variables

Demographic data and parameters related to heart failure were obtained from the SwedeHF. Demographic data included gender, age at first hospitalisation for heart failure and comorbidities. Parameters related to heart failure included primary aetiology, LVEF, results from blood samples (eg, N-terminal pro B-type natriuretic peptide (NTproBNP) at admission and discharge), NYHA class, medication, device therapy, etc. Relevant dates were included, such as date of hospital discharge and date of death obtained from this registry.

The date of cardiac arrest was obtained from the SRCR. The primary outcome was cardiac arrest (OHCA and IHCA) after hospital discharge. The date of final follow-up for cardiac arrests registered in SRCR was 15 November 2022.

### Statistical analysis

Baseline characteristics are presented as count (%) for categorical variables and mean (SD) for continuous variables. The baseline characteristics are presented with the study population stratified into four groups. Group 1 includes the entire study population. Group 2 includes patients who had a cardiac arrest treated with CPR registered in SRCR. Group 3 consists of patients who died without receiving CPR. Lastly, group 4 consists of patients who had no event, in other words were censored, by the end of the study.

**Figure 1:**
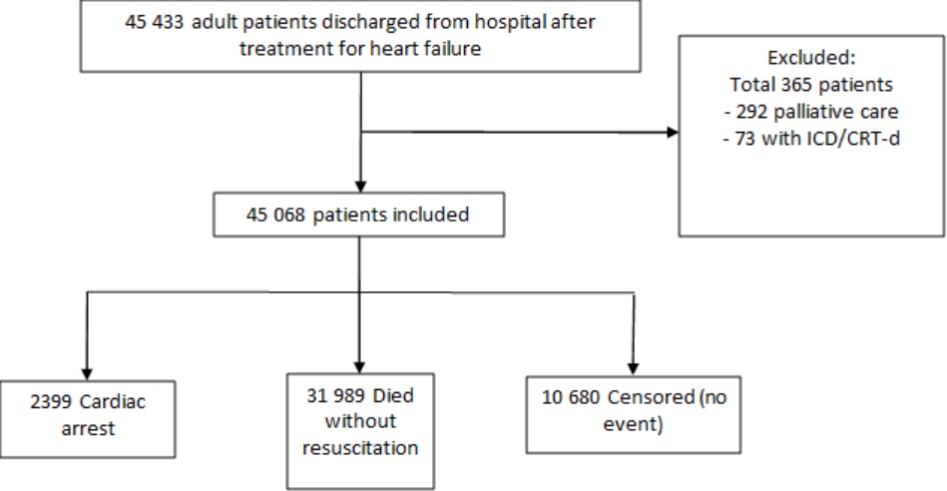
Flowchart of the three possible patient outcomes for patients discharged from hospital after first hospitalisation for heart failure. The primary outcome in this study is cardiac arrest registered in the Swedish Registry for Cardiopulmonary Resuscitation (SRCR). Death without resuscitation is a competing event for cardiac arrest, these are patients who died but were not registered in the SRCR. Censored are the patients who survived to the final follow-up date, 15 November 2022, without any of the aforementioned events. CRT-d, cardiac resynchronisation therapy with defibrillator; ICD, implantable cardioverter defibrillator.

Death without CPR was considered a competing event for cardiac arrest treated with CPR registered in SRCR, because a deceased individual is unable to suffer from a cardiac arrest treated with CPR thereafter. As presented in figure 1, the patients included in this study population had three possible outcomes, cardiac arrest according to SRCR, death without resuscitation and finally those who had no event by the final follow-up date (15 November 2022), meaning they were censored.

Cumulative incidence functions for cardiac arrest and death without resuscitation as first events were estimated using the Aalen-Johansen estimator, accounting for the competing risk structure. The resulting cumulative incidence curves are presented as step functions reflecting changes at observed event times.

Missing data were handled using data imputation with MissRanger package in R, based on the existing data patterns. MissRanger uses a Random Forest algorithm to predict the missing values while maintaining the original data distribution.

Due to a competing event for cardiac arrest, we built a prediction model using the Random Survival Forest algorithm for competing risk based on training data (70% of the dataset). All available variables from the SwedeHF were used as predictors, except continuous variables of prescribed doses of heart failure medications. These variables were excluded due to a high degree of missingness. We used the RandomForestSRC package in R. This method of building a prediction model uses a combination of multiple survival trees grown for each competing event.^19^ We used cross-validation with fivefolds and one repeat in the training process. A manual hyperparameter grid search was performed. The model with the best performing set of hyperparameters was evaluated using the test data. The model performance is evaluated using the Brier score, observed versus the model’s predicted cumulative incidence, Concordance index (C-index) and time-dependent area under the curve (AUC) of a receiver operating characteristics (ROC) graph. Brier score is a metric for a model’s calibration, which measures the model’s capacity to predict the group level risk for the event such as cumulative incidence. A lower Brier score is indicative of a high model calibration. Similarly, plotting the observed and the model’s predicted cumulative incidence visualises the model’s capacity to predict cumulative incidence. C-index and time-dependent AUC-ROC are performance metrics that measure the model’s capacity to predict the primary event in each individual case. Variable importance is presented for the 10 most important variables, and the partial-dependency plots were plotted for these same 10 variables. Partial-dependency plots visualise how levels of a categorical variable or values of a continuous variable are associated with years lost to the primary event.

All analyses were performed using R V.4.4.2 (R Foundation for Statistical Computing, Vienna Austria).

### Patient and public involvement

None.

## Results

### Baseline characteristics

A total of 45 433 patients were registered in the SwedeHF. 356 patients were excluded based on criteria (figure 1). As presented in the baseline table (table 1), of the 45 068 patients included, 2399 (5.3 %) were registered in SRCR for cardiac arrest where CPR was initiated. A majority of the remaining patients (70.9%) had died without resuscitation; these are patients who were not registered in the SRCR. Lastly, 10 680 (23.7%) patients were censored with no event by the final follow-up. The average number of years to event was similar in the patient group with cardiac arrest and the patients who died without resuscitation (mean (SD) 2.7 (3.0) and 2.6 (3.0), respectively). The average number of years to last follow-up date was 6.7 (4.3) (mean (SD)) for the patients who had no event. The average age of patients who had died without resuscitation was mean (SD) 80.7 years (9.5). Patients who were censored with no event had an average age of mean (SD) 68.1 years (13.3), and patients with cardiac arrest had an average age of mean (SD) 73.8 years (11.2). A majority of patients who had cardiac arrest with CPR were men 68.7%, and the group of patients who had died without resuscitation almost had an even distribution of the sex, with 53.3% men. A majority of the patients who died without resuscitation had some difficulty with mobility (60.6%). 51% of patients who suffered from cardiac arrest had some difficulty with mobility. Average levels of NTproBNP at hospital discharge were mean 8860.6 pg/mL in patients who had died without resuscitation and mean 7052.1 pg/mL in the patients who had a cardiac arrest. Estimated glomerular filtration rate (e-GFR) at hospital admission and hospital discharge was on average 71.36 and 67.6 mL/min/1.73 m^2^ in patients who were censored with no event, mean 71.36 and 67.6 mL/min/1.73 m^2^ in patients with cardiac arrest and 56.5 and 54.0 mL/min/1.73 m^2^ in the patient group who had died without resuscitation (table 1).

**Table 1:** Baseline characteristics

	Overall	Cardiac arrest	Death without resuscitation	Censored (no event)	Missingness
n	45 068	2399	31 989	10 680	
Number of years to last follow-up date or first event (mean (SD))	3.6 (3.8)	2.7 (3.0)	2.6 (3.0)	6.7 (4.3)	0.0
Age (mean (SD))	77.4 (11.9)	73.8 (11.2)	80.7 (9.5)	68.1 (13.3)	0.0
Sex, male (%)	25 236 (56.0)	1647 (68.7)	17 052 (53.3)	6537 (61.2)	0.0
Hypertension (%)	25 535 (57.8)	1410 (59.8)	18 407 (58.9)	5718 (54.1)	1.9
Chronic lung disease (%)	8590 (19.5)	558 (23.8)	6610 (21.2)	1422 (13.5)	2.2
Diabetes mellitus (%)	9772 (21.7)	807 (33.6)	7759 (24.3)	1206 (11.3)	0.0
Atrial fibrillation	24 371 (54.4)	1157 (48.5)	18 258 (57.4)	4956 (46.6)	0.5
Earlier myocardial infarction (%)	9120 (31.5)	581 (40.1)	6334 (34.4)	2205 (24.3)	35.8
Previous coronary revascularisation (%)	11 416 (25.3)	793 (33.1)	7986 (25.0)	2637 (24.7)	2.0
Pre-existing valve disease (%)	9843 (22.6)	524 (22.5)	7488 (24.5)	1831 (17.3)	3.5
Previous valve surgery (%)	2895 (6.4)	160 (6.7)	2098 (6.6)	637 (6)	0.7
Primary aetiology (%)					49.4
Alcohol	143 (0.6)	11 (0.9)	57 (0.4)	75 (1.1)	
Dilated cardiomyopathy	892 (3.9)	42 (3.6)	325 (2.2)	525 (7.5)	
Valve disease	2153 (9.4)	90 (7.7)	1490 (10.2)	573 (8.2)	
Hypertension	6861 (30.1)	317 (27.0)	4693 (32.1)	1851 (26.5)	
Ischaemic heart disease	8265 (36.2)	527 (45.0)	5466 (37.3)	2272 (32.5)	
Other	4490 (19.7)	185 (15.8)	2606 (17.8)	1699 (24.3)	
NYHA (%)					56.4
NYHA I	2109 (10.7)	108 (9.3)	1220 (8.7)	781 (17.6)	
NYHA II	8014 (40.8)	498 (42.9)	5402 (38.5)	2114 (47.7)	
NYHA III	8081 (41.2)	492 (42.4)	6230 (44.4)	1359 (30.7)	
NYHA IV	1426 (7.3)	62 (5.3)	1189 (8.5)	175 (4.0)	
Level of mobility (%)					84.8
Bedridden	243 (3.6)	10 (3.0)	202 (5.4)	31 (1.1)	
Some difficulty	3513 (51.3)	168 (51.2)	2259 (60.6)	1086 (38.9)	
Independent	3088 (45.1)	150 (45.7)	1264 (33.9)	1674 (60.0)	
Left bundle branch block	6356 (16.5)	376 (18.3)	4418 (16.5)	1562 (16.0)	14.5
Left ventricular ejection fraction (%)					23.3
Normal, ≥50%	10 216 (29.5)	500 (25.0)	7869 (32.4)	1847 (22.2)	
Mild, 40%–49%	7161 (20.7)	393 (19.6)	5104 (21.0)	1664 (20.0)	
Moderate, 30%–39%	8270 (23.9)	552 (27.6)	5617 (23.2)	2101 (25.2)	
Severe, <30%	8930 (25.8)	558 (27.9)	5662 (23.3)	2710 (32.6)	
NTproBNP at hospital discharge, pg/mL (mean (SD))	6398.5 (9106.1)	7052.1 (9304.0)	8860.6 (11 125.5)	4635.7 (6841.3)	93.3
BNP at hospital discharge, pg/mL (mean (SD))	3742.7 (7138.5)	9861.3 (9995.3)	5528.3 (9685.4)	1469.5 (1679.4)	99.9
e-GFR at hospital admission, mL/min/1.73 m^2^ (mean (SD))	60.3 (28.5)	62.0 (32.7)	56.5 (27.0)	71.36 (29.2)	0.5
e-GFR at hospital discharge, mL/min/1.73 m^2^ (mean (SD))	61.3 (35.4)	59.6 (25.7)	54.0 (30.3)	67.6 (38.6)	83.4
Heart failure duration <6 months (%)	20 766 (52.8)	1180 (53.4)	14 134 (48.0)	5452 (71.0)	12.7
Device therapy (%)					0.0
CRT	381 (0.8)	31 (1.3)	283 (0.9)	67 (0.6)	
CRT-D or ICD	0 (0.0)	0 (0.0)	0 (0.0)	0 (0.0)	

BNP, brain natriuretic peptide; CRT, cardiac resynchronisation therapy; CRT-D, cardiac resynchronisation therapy with defibrillator; e-GFR, estimated glomerular filtration rate; ICD, implantable cardioverter defibrillator; NTproBNP, N-terminal pro B-type natriuretic peptide; NYHA, New York Heart Association.

### Event cumulative incidence

**Figure 2:**
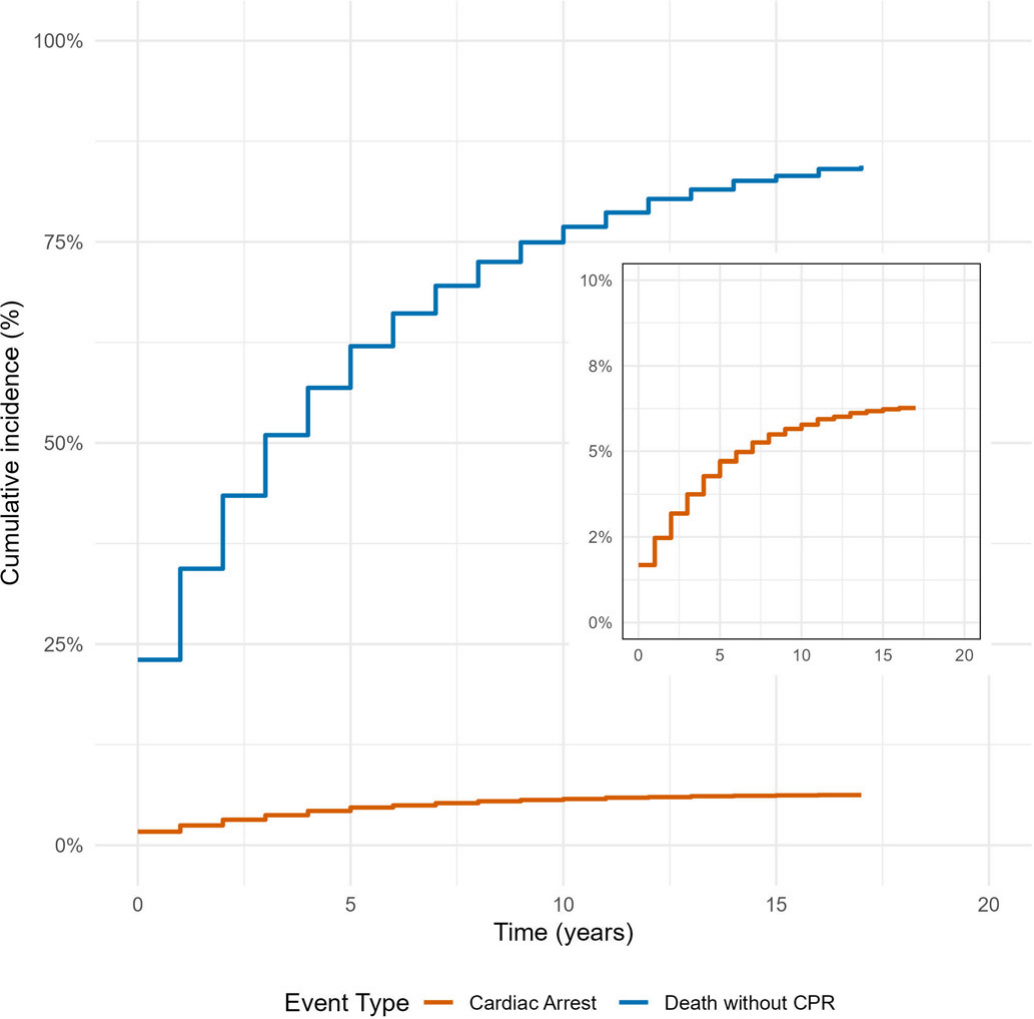
Cumulative incidence for cardiac arrest and death without resuscitation in the study population of patients discharged after first hospitalisation for heart failure. Cumulative incidence functions were estimated using the Aalen-Johansen estimator and are displayed as step functions reflecting observed event times. Including a zoomed inset plot of the cumulative incidence of cardiac arrest. CPR, cardiopulmonary resuscitation.

The cumulative incidence of cardiac arrest increased gradually over time and remained below 8% throughout follow-up, with a tendency to plateau after approximately 10 years. In contrast, the cumulative incidence of death without resuscitation increased substantially over time, exceeding 75% at 10 years (figure 2). This marked difference in event frequency indicates that death without resuscitation was the dominant outcome in the cohort.

### Model performance evaluation

**Figure 3:**
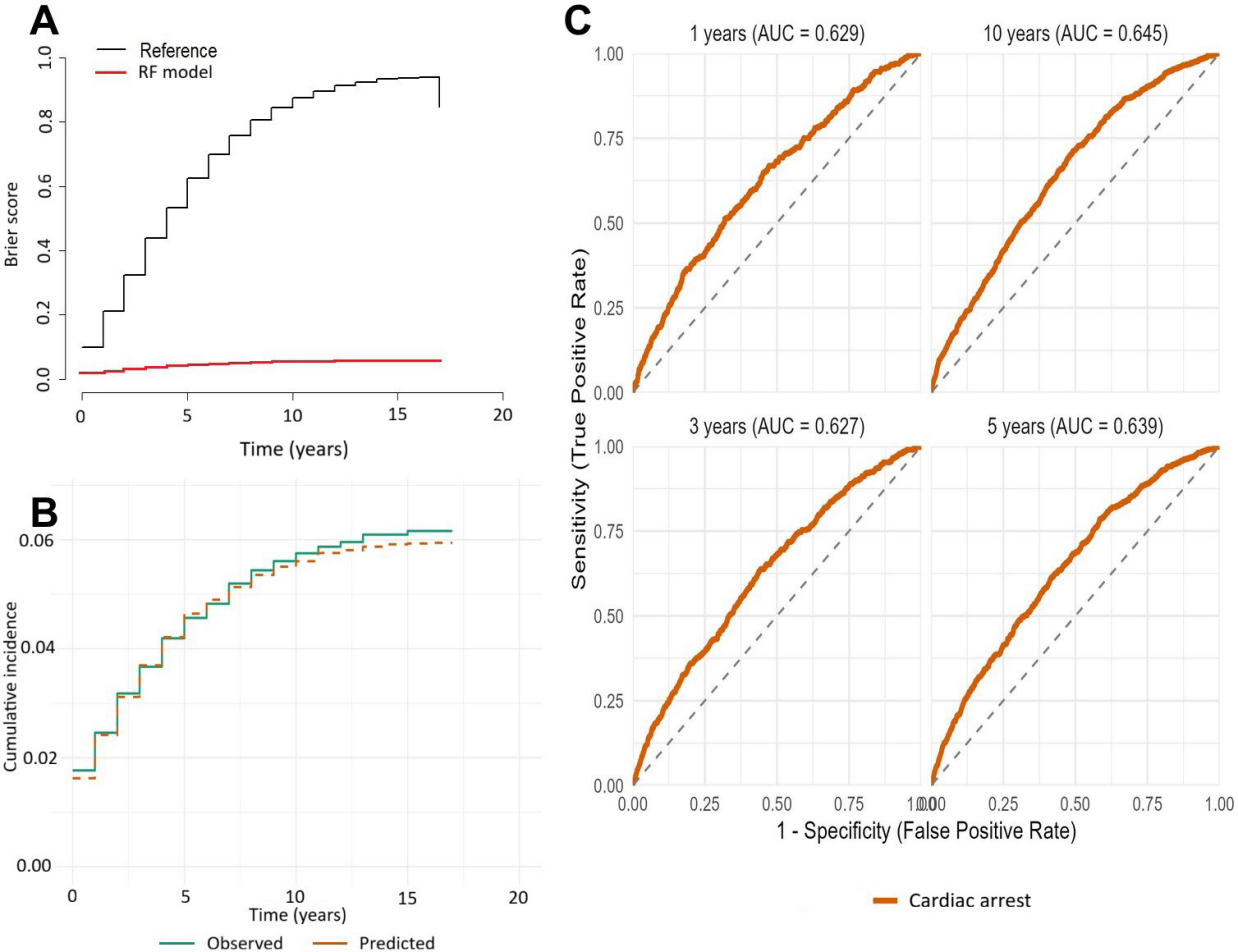
Evaluation of performance of Random Survival Forest for competing risk predicting cardiac arrest versus death without resuscitation or no event with 82 predictors. (A) Brier score over time. (B) Observed cumulative incidence and model predicted cumulative incidence of cardiac arrest over time. (C) Receiver operating characteristics graph of Random Forest model predicting cardiac arrest with time-dependent area under the curve at 1, 3, 5 and 10 years after inclusion. AUC, area under the curve; RF model: Random Forest model.

In figure 3, we present the performance metrics of our Random Survival Forest model for competing risk with a total of 82 variables. Definitions for each included variable can be found in online supplemental table S1. Plot A visualises the Brier score, and plot B visualises the model’s predicted and observed cumulative incidence over time. Brier score was low, and predicted cumulative incidence matched well with the observed cumulative incidence. The time-dependent ROC graphs present the model’s capacity to predict the cardiac arrest outcome versus all other outcomes at different time points. The AUC of these ROC curves ranged from 0.63 to 0.65 at year 1, 3, 5 and 10 after hospital discharge. The model’s C-index for predicting cardiac arrest was 0.52.

### Variable importance

**Figure 4:**
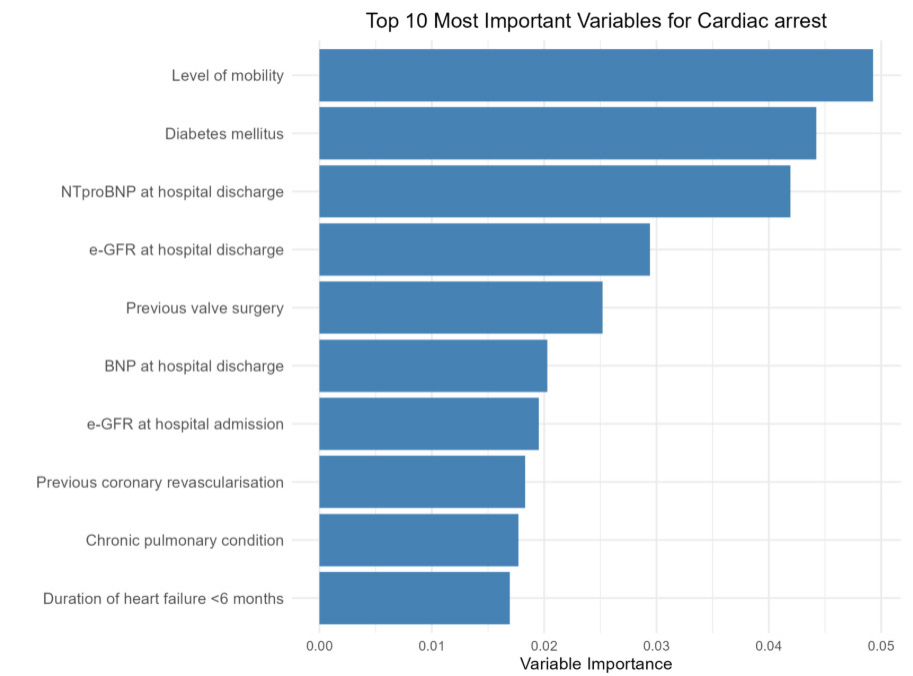
Variable importance of the 10 most predictive variables from the Random Survival Forest model for competing risk predicting cardiac arrest vs death without resuscitation or no event. e-GFR, estimated glomerular filtration rate; NTproBNP, N-terminal pro B-type natriuretic peptide.

**Figure 5:**
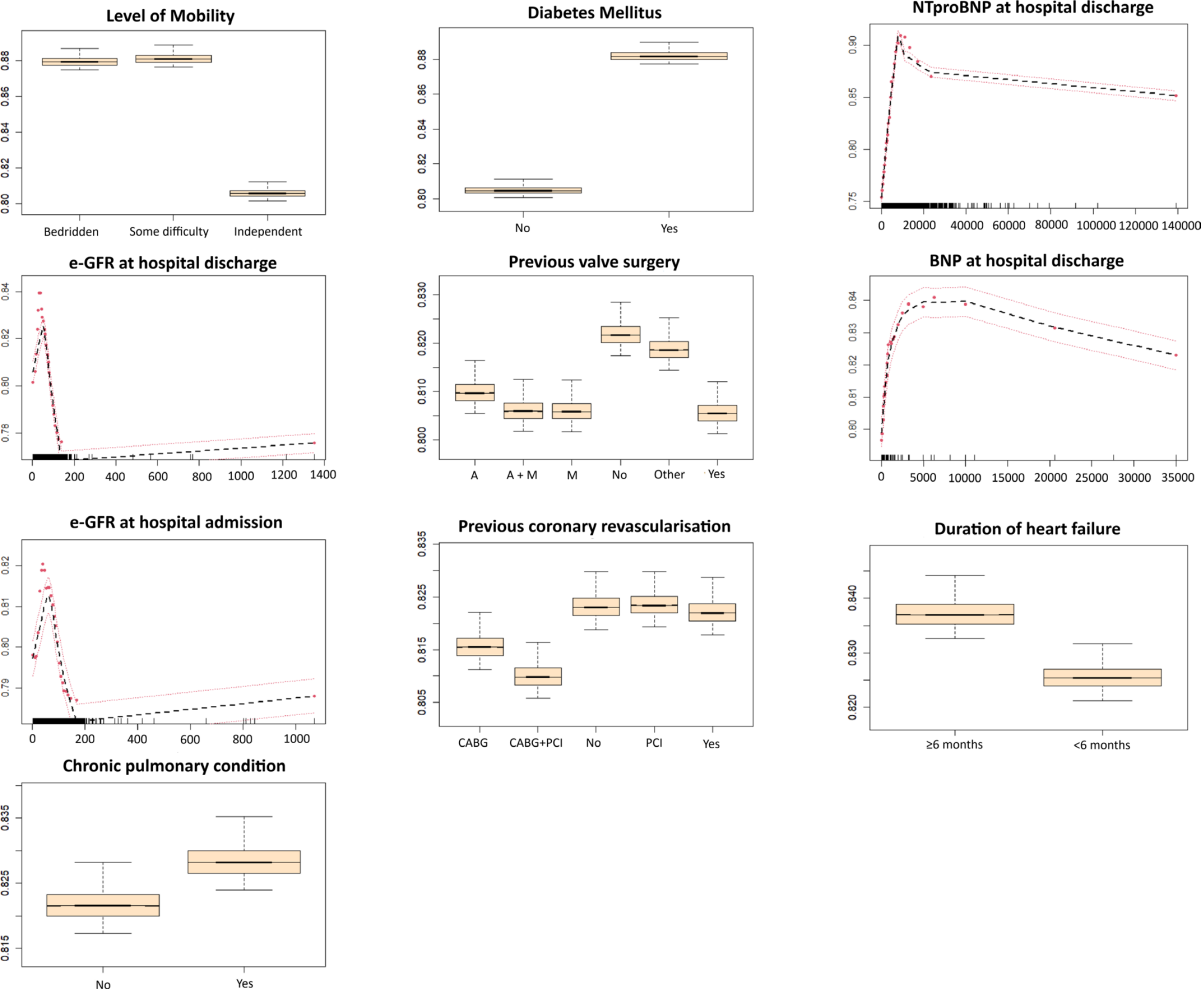
Partial dependency plots for the top 10 variables, plotted against years lost to cardiac arrest. A, aortic valve surgery; A+M, aortic and mitral valve surgery; BNP, B type natriuretic peptide; CABG, coronary artery bypass graft surgery; e-GFR, estimated glomerular filtration rate; M, mitral valve surgery; No, no valve surgery; NTproBNP, N-terminal pro-B-type natriuretic peptide; Other, other valve surgery; PCI, percutaneous coronary intervention; Yes, unspecified valve surgery.

Among the 10 variables with greatest importance for the model’s prediction, level of mobility, NTproBNP at hospital discharge after heart failure diagnosis and presence of pre-existing diabetes mellitus were the top 3. This was followed by e-GFR at hospital discharge and previous valve surgery (figure 4). In figure 5, partial dependency plots show how the model interprets the association between the 10 variables with greatest importance and predicted years lost to cardiac arrest. Increased difficulty with mobility, diabetes, chronic pulmonary conditions and duration of heart failure condition>6 months were associated with increased predicted years lost to cardiac arrest, in other words increased predicted risk of cardiac arrest. The absence of valve surgery, absence of revascularisation or percutaneous coronary intervention were also associated with more predicted years lost to cardiac arrest. Initially increasing levels of NTproBNP at hospital discharge and BNP at hospital discharge were associated with more predicted years lost to cardiac arrest. However, this trend changed after a point as further increase in these parameters were associated with decreasing predicted years lost to cardiac arrest. e-GFR rate at hospital admission and hospital discharge also had a non-linear relationship with years lost to cardiac arrest. Low e-GFR was associated with fewer predicted years lost to cardiac arrest, moderate-high e-GFR showed an increase in predicted years lost to cardiac arrest and even higher e-GFR was associated with almost no years lost to cardiac arrest.

## Discussion

In this study we developed a machine learning model using Random Survival Forest for competing risk to predict cardiac arrest in patients discharged from hospital after their first hospitalisation for newly diagnosed heart failure. Cardiac arrest was defined as cardiac arrests where CPR was initiated and registered in the SRCR. Death without resuscitation was a competing event. Using predictors obtained from the SwedeHF, this model had a poor capacity to predict the outcome cardiac arrest in each individual case as C-index and AUC-ROC were almost 0.5, yet the model calibration was excellent. This means that the model is able to predict the risk for cardiac arrest on a group level but is no better than random guessing when it comes to discriminating between cardiac arrest and any other event (death without resuscitation and censored) for each individual.

Variables in the registry for heart failure were related to the severity of the heart failure condition. The variables of greatest importance represent the variables relating to heart failure that the model primarily used to predict the cumulative incidence on a group level and predict the primary event on an individual level. Level of mobility was the variable with greatest importance, suggesting that some degree of diminished mobility seems to be associated with cardiac arrest. This could in part be due to the effect of mobility on survival or the influence that poor mobility has on the overall health status.^20 21^ Elevated levels of NTproBNP, diabetes and kidney failure in patients with heart failure are known to be associated with sudden cardiac arrest and all-cause mortality. Thus, it is unsurprising that the model finds these predictors to be of great importance for predicting the cumulative incidence of cardiac arrest. Previous findings show that the important predictors for our model are associated with sudden cardiac arrest as well as all-cause mortality.^22^,^29^ Hence why, our model is well calibrated to predict the cumulative incidence on a group level.

Cardiac arrest is part of the dying process even in the context of palliation. Patients who are deemed to benefit from CPR in the event of an unexpected cardiac arrest are those who could benefit from preventative measures. We reasoned that it is more likely that these are the patients included in the SRCR compared with patients who died without resuscitation. Patients with Do-Not-Attempt-Cardiopulmonary-Resuscitation-orders (DNACPR-orders) due to poor health conditions likely belong to the group who died without resuscitation. The average age of this patient group being higher and level of mobility being lower than the other patient groups may indicate that many of these patients belong to an elderly population who are often allowed a natural death without intervention.^30^,^32^ Furthermore, the trend of decreasing years lost to cardiac arrest at high levels of BNP or NT-proBNP at hospital discharge in the partial dependency plots could possibly be explained by these patients having palliative care and passing away without resuscitation. A low e-GFR at hospital admission and discharge show less life is lost to cardiac arrest, these patients may have died without CPR (due to DNACPR-orders). With improved eGFR, these patients may have become eligible for CPR and are more likely to experience cardiac arrest with CPR, so predicted years lost to cardiac arrest rises. After a certain threshold, better kidney function might become protective, reducing the incidence of cardiac arrest or death, so the patients were more likely to be censored, and years lost declines. However, it is not registered in our data whether any of these patients had died in palliative care. We do not have data on transition to palliative care after discharge from the first hospitalisation. This is likely the reason for the poor discriminatory capacity of this model. Ideally, patients who transitioned to palliative care or received DNACPR-orders should be excluded from observation from then on. In such a case, a composite endpoint of cardiac arrest in SRCR and death without resuscitation would include all cardiac arrests eligible for CPR and preventative measures, regardless of whether the patient actually received CPR. Such an outcome would represent the biological event of cardiac arrest which we had primarily intended to predict. But the current model is in fact predicting cardiac arrests treated with CPR, a non-biological event.

### Limitations

There are limitations in our model and if the training was adjusted it could improve performance, such as having a higher number of decision trees (>1000) and more repeats in the cross-validation. Yet, we find that there are fundamental limitations in our data that limit the performance of this model.

The model is constructed to predict cardiac arrest several years after the first hospitalisation for heart failure. But the model includes predictors from this first hospitalisation which may not be relevant for the prediction of a cardiac arrest event several years later. Additional updated data on the patient status from hospitalisations during the follow-up time would improve clinical relevance.

A cardiac arrest is more likely to be reported to SRCR if it was witnessed, if bystander CPR was initiated, and emergency medical services were alerted. Furthermore, the logistics in case-reporting should work as well. There is a certain randomness/unpredictability to these non-biological circumstances which a machine learning model using biological heart failure related parameters cannot predict. Furthermore, previous studies have shown that SRCR is incomplete.^17 18^ These missing cases could be misclassified in our study as deaths without resuscitation or censored with no event. Even a small proportion of in-hospital deaths are a result of CPR not being performed despite there not being a DNACPR-order.^33^ Thus, the group of patients who died without resuscitation may have included patients who should have been treated with CPR and based on our definition could have benefitted from preventive measures. We were able to define our competing events, but limitations in the data result in mixed patient groups not adhering to these distinct event definitions.

### Future perspectives

The potential to accurately predict cardiac arrest in each individual case has been made possible with the introduction of artificial intelligence and machine learning.^34^ This has the potential to give a focused approach to preventive treatment. Currently previously published studies on machine learning models developed for cardiac arrest prediction are based on dynamic time-sensitive physiological data such as vital signs, ECG, etc .^12 35 36^ But our approach uses static, baseline data and known clinical risk factors to develop a machine learning prediction model. If such a model performed well and superior to existing risk models, it would be clinically useful for risk stratification and improved resource allocation. Through the years much of cardiac arrest research into survival has been supplied by the data from registries with robust quality and impressive sample size.^37 38^ The results of this study suggest that the requirements of research into prediction of cardiac arrest cannot be met by the data from our registries. Primarily, Swedish registries are missing vital data on DNACPR-orders and data regarding palliative care. These treatment strategies are documented in the medical records. Thus, data collection should be possible and is evidently crucial for the objective we had in this study as these patients should have been excluded.

## Conclusion

Our Random Survival Forest model for competing risk could not accurately predict cardiac arrest in individual patients newly diagnosed with heart failure, because the event death without attempted resuscitation was treated as a competing event. The lack of information on transitions to palliative care and Do-Not-Attempt-CPR-orders limits the clinical relevance of any cardiac arrest prediction model.

## Supplementary material

10.1136/bmjopen-2025-113890 online supplemental file 1

## Data Availability

No data are available.
