# Supplementary material for: Prediction of cardiac arrest in patients with heart failure in Sweden: a registry study with development of a machine learning model
Source: BMJ Open. 2026 Jun 25;16(6):e113890. doi: 10.1136/bmjopen-2025-113890 (PMC13311599; doi:10.1136/bmjopen-2025-113890)
Supplement: online supplemental file 1 [file bmjopen-16-6-s001.docx]

## Supplementary material

**Table S1: Definitions of all included predictors in the model**

| **Predictor** | **Definition** |
| --- | --- |
| Serial number | Unique patient serial number |
| Sex | Biological sex male/female |
| Age | Age as continuous variable |
| Type | Type of registration, index registration, follow-up or yearly-followup. (All registrations were index) |
| Reg_step | Registration step, follow-up or index from open clinic or hospital ward. (all registrations were index registrations from hospital ward) |
| Duration | Estimated duration of the heart failure, ≥6 months or <6 months |
| Alcohol_f | How often do you, as a woman, drink 4 standard glasses and if you are a man, 5 standard glasses or more on one occasion? “Daily/nearly daily”, “every month”, “every week”, “less than 1/month or never” |
| Alcohol_vol | How many standard glasses do you drink in a typical week?  “≥15 standard glass/week”, “10-14 standard glass/week”, “5-9 standard glass/week”, “1-4 standard glass/week”, ”<1 standard glass/week” |
| Anxiety | How often do you experience anxiety?  “Often anxious/sad”, “sometimes anxious/sad”, “Never anxious/sad” |
| Fatigue | Description of fatigue: “Fatigue in rest”, “Fatigue at mild exertion”, “Fatigue at moderate exertion”, “Never fatigued” |
| Hygiene | Description of personal hygiene: “Receives aid in personal hygiene”, “Receives aid in personal hygiene”, “No help with personal hygiene” |
| Quality of life score | Numeric score from EuroQol Visual Analogue Scale |
| Alcohol | Degree of alcohol consumption: "Ongoing/prior problematic drinking", "Ongoing/prior problematic drinking", "Normal drinking habits", "Never" |
| Activity | Degree of activity: "Receives aid in main acitivities", "Receives aid in main activities", "Can manage main activities" |
| Mobility | Degree of mobility: "Bedridden", "Can walk but with some difficulty", "No problems walking" |
| Pain | "No pain", "Severe/moderate pain", "Severe/moderate pain" |
| HF_school | Taken part in heart failure school |
| HF_training | Taken part in physiotherapy for heart failure |
| Dyspnea | "Dyspnea at rest", "Dysnpea at moderate exertion", "Dyspnea at mild exertion", "No dyspnea" |
| Smoking | "Never smoked", "Daily smoking/prior smoker/smokes, but not daily", "Daily smoking/prior smoker/smokes, but not daily", "Daily smoking/prior smoker/smokes, but not daily", "Daily smoking/prior smoker/smokes, but not daily", "Daily smoking/prior smoker/smokes, but not daily" |
| Atrial fibrillation | History of atrial fibrillation |
| Chronic lung disease | History of chronic lung disease |
| Diabetes | History of diabetes |
| Dilated cardiomyopathy | Historu of dilated cardiomyopathy |
| Earlier Myocardial infarction | History of myocardial infarction |
| Valve disease | History of heart valve disease |
| Valve surgery | History of valve surgery |
| Hypertension | History of hypertension |
| Primary etiology | Primary aethiology for heart failure: "Alcohol", "dilated kardiomyopathy", "valve disease", "hypertension", "Ischemic heart disease", "Other" |
| Revascularization | History of coronary revascularisation: |
| BNP_admission | Continuous value of B-type natriuretic peptide (BNP) at hospital admission |
| BNP_Discharge | Continuous value of B-type natriuretic peptide (BNP) at hospital discharge |
| dBP_admission | Diastolic blood pressure at hospital admission |
| dBP_discharge | Diastolic blood pressure at hospital discharge |
| sBP_admission | Systolic blood pressure at hospital admission |
| sBP_discharge | Systolic blood pressure at hospital discharge |
| Hb admission | Hemoglobin level at hospital admission |
| Hb discharge | Hemoglobin level at hospital discharge |
| e-gfr admission | Estimated glomerular filtration rate at hospital admission |
| e-gfr discharge | Estimated glomerular filtration rate at hospital discharge |
| NYHA | New York Heart association functional classification |
| Heart frequency admission | Herat frequency at hospital admission |
| Heart frequency discharge | Heart frequency at hospital discharge |
| Height | Height in centimeters |
| Killip class | Killip class at hospital admission |
| NTproBNP admission | N-terminal pro-B-type natriuretic peptide at hospital admission |
| NTproBNP discharge | N-terminal pro-B-type natriuretic peptide at hospital discharge |
| Transferrin_admission | Level of transferrin in serum at hospital admission |
| Creatinine_Admission | Level of creatinine in serum at hospital admission |
| Creatinine_discharge | Level of creatinine in serum at hospital discharge |
| Ferritin_admission | Level of Ferritin in serum at hospital admission |
| Potassium_admission | Level of potassium in serum at hospital admission |
| Potassium_Discharge | Level of potassium in serum at hospital discharge |
| Sodium admission | Level of sodium in serum at hospital admission |
| Sodium discharge | Level of sodium in serum at hospital discharge |
| Weight | Body weight in kilograms |
| Chest-x-ray | Chest-x-ray results at hospital admission: "Enlarged heart", "No", "Normal", "Pulmonary stasis", "Pulmonary stasis + enlarged heart" |
| ECG | Electrocardiogram results at hospital admission: "Atrial fibrillation", "Other rhythm", "Pacemaker", "Sinusrhythm" |
| Left bundle branch block | Presence of left bundle branch block |
| Left ventricular ejection fraction method | Method used to measure Left ventricular ejection fraction (LVEF): "ultrasound cardiography", "Magnetic Resonance Imaging", "Not applicable", "Scintigraphy" |
| LVEF value | LVEF value as continuous variable |
| LVEF factor | LVEF value as categorical variable: "Mild, 40-49%", "Moderate, 30-39%", "Normal, ≥50%", "Severe, <30%" |
| QRS width | Width of QRS-complex in ECG |
| ARB | Administered an angiotensin-receptor II blocker during hospital stay or at hospital discharge |
| ACE inhibitor | Administered an angiotensin-converting enzyme inhibitors during hospital stay or at hospital discharge |
| Anticoagulant | Administered an anticoagulant during hospital stay or at hospital discharge |
| ARNI | Administered an Angiotensin-receptor-Neprilysin inhibitor during hospital stay or at hospital discharge |
| Antiplatelet | Administered an antiplatelet medication during hospital stay or at hospital discharge |
| Beta blocker | Administered a beta-blocker during hospital stay or at hospital discharge |
| Device therapy | Discharged from hospital with device therapy: "CRT", "CRT-D", "ICD", "No", "Pacemaker" |
| Digoxin | Administered Digoxin during hospital stay or at hospital discharge |
| Ferrocarboxy | Administered ferric carboxymaltose during hospital stay or at hospital discharge |
| Inotrope | Administered an Inotropic drug during hospital stay or at hospital discharge |
| Long nitrate | Administered a loop nitrate during hospital stay or at hospital discharge |
| Loop diuretic | Administered a loop-diuretic during hospital stay or at hospital discharge |
| Loop diuretic usage | How loop-diuretic was administered, daily dose or when necessary. |
| MRA | Administered a Mineralocorticoid receptor antagonists during hospital stay or at hospital discharge |
| SGLT2 | Administered a SGLT2-inhibitor during hospital stay or at hospital discharge |
| Sinus node inhibitor | Administered a sinus-node inhibitor during hospital stay or at hospital discharge |
| Statin | Administered a Statin during hospital stay or at hospital discharge |
| Palliative | Discharge from hospital with palliative care |
| BMI | Body Mass Index |
