# Supplementary material for: Prediction of cardiac arrest in patients with heart failure in Sweden: a registry study with development of a machine learning model
Source: BMJ Open. 2026 Jun 25;16(6):e113890. doi: 10.1136/bmjopen-2025-113890 (PMC13311599; doi:10.1136/bmjopen-2025-113890)
Supplement: Supplementary file 2 [file bmjopen-2025-113890.reviewer_comments.pdf]

PEER REVIEW HISTORY

BMJ Open publishes all reviews undertaken for accepted manuscripts. Reviewers are asked to complete a checklist review form and are provided with free text boxes to elaborate on their assessment. These free text comments are reproduced below.

ARTICLE DETAILS

Title (Provisional)

Prediction of cardiac arrest in patients with heart failure in Sweden – a registry study with development of a machine learning model

Authors

Thuccani, Meena; Rawshani, Araz; Rylander, Christian; Herlitz, Johan; Lundgren, Peter

---

VERSION 1 - REVIEW

**Reviewer**	**1**
**Name**	**Hikoso, Shungo**
**Affiliation**	**Nara Medical University, Department of Cardiology**
**Date**	**22-Jan-2026**
**COI**	**None**

This study addresses an important clinical question and applies a competing risk framework to predict cardiac arrest in patients with newly diagnosed heart failure. The use of cumulative incidence functions is appropriate and adds value compared with conventional approaches. However, several aspects related to outcome definition, prediction time horizon, variable transparency, and presentation currently limit the interpretability and clinical applicability of the results. I believe that further clarification and additional analyses are needed according to following comments..

Major comments

1. The primary outcome is defined as cardiac arrest treated with CPR, while death without resuscitation is treated as a competing event. As acknowledged by the authors, death without CPR likely includes a substantial proportion of patients with DNACPR orders or those who transitioned to palliative care after discharge. In this setting, the competing event may largely reflect treatment decisions rather than true biological competing risk, which limits the interpretation of the model as predicting intrinsic cardiac arrest risk. To address this limitation more constructively, the authors may consider alternative or expanded outcome definitions. If registry data are available, inclusion of appropriate ICD or CRT-D therapies (e.g., appropriate shocks for ventricular tachyarrhythmias) as part of a composite outcome

representing “aborted sudden cardiac death” could be explored. Such an approach may reduce dependence on CPR initiation and better capture clinically meaningful malignant arrhythmic events. At minimum, the feasibility and potential impact of this alternative outcome definition should be discussed, and sensitivity analyses using different outcome definitions could strengthen the clinical relevance of the findings.

2. The current analysis evaluates outcomes over a long follow-up period of up to 10 years after discharge. For prediction of sudden cardiac arrest in heart failure, such a long time horizon may be strongly influenced by changes in clinical status, treatment, and goals of care over time, potentially diluting clinically relevant signals. To improve clinical interpretability, the authors may consider performing separate prediction analyses for clearly defined short-term horizons, such as 3, 6, and 12 months after discharge. Although the number of events would be smaller, these time frames may better reflect periods in which baseline clinical information is most relevant and competing risks related to treatment decisions are less dominant. Presenting model performance across these distinct time windows would help clarify whether predictive ability differs by follow-up duration.

3. The manuscript states that 82 predictors were included in the model, but Table 1 presents only a limited subset of baseline variables. It is currently unclear which variables were included in the model, at what time points they were measured (admission or discharge), and how much missing data was present for each predictor. Providing a complete list of predictors with basic information (definition, timing, and missingness), preferably as supplementary material, would substantially improve transparency and reproducibility.

4. Some predictors appear to represent closely related or repeated measurements (for example, admission and discharge values of similar laboratory parameters). While tree-based models can accommodate correlated variables, consideration of a more parsimonious predictor set, or sensitivity analyses using a reduced and clinically standard variable set, may improve interpretability and generalizability of the model.

5. The figures could be refined to better support interpretation of the results. In particular, Figure 2 would benefit from clearer visualization of the cumulative incidence functions and clarification of how time points were handled in estimation and plotting. Given the large sample size, it would be helpful to explain whether the stepwise appearance of the curves reflects the estimation method or time discretization. Minor improvements in figure presentation would enhance readability.

6. The overall narrative could be strengthened by more clearly aligning the study objective, analytical approach, and interpretation. Explicitly stating whether the primary aim is biological risk prediction of sudden cardiac arrest or prediction of CPR-treated cardiac arrest would help readers better understand the scope and limitations of the study.

**Reviewer**	**2**
**Name**	**Yagdi, Tahir**

**Affiliation**	**Ege Universitesi**
**Date**	**07-Mar-2026**
**COI**	**None**

I carefully read the article titled “Prediction of cardiac arrest in patients with heart failure – development of a machine learning model" sent to me for review. My comments, criticisms, and suggestions are listed below:

1. According to the literature, third-degree AV block and left bundle branch block are considered significant predictors of sudden cardiac arrest. Do the authors have any findings regarding this? A discussion on this topic would be appropriate. Similarly, the impact of the presence of paroxysmal or long-term atrial fibrillation should be evaluated.

2. Possible mechanisms of sudden cardiac arrest include left ventricular hypertrophy, neurohormonal activation, myocardial ischemia, and cardiac fibrosis. Have these findings been analyzed? I would appreciate comments on this.

3. Can any comments be made regarding the impact of heart transplantation and mechanical circulatory support system implantation, which are the gold standard surgical treatment methods in the surgical treatment of end-stage heart failure, on the outcomes?

4. Have the impacts of the number and frequency of cardiac-related hospital admissions been evaluated? Have the changes in treatment during these admissions been assessed? Have any potential changes affected outcomes? I suggest this for discussion.

5. It would be beneficial to discuss, in light of the literature, the impact of changes over the years on the results of parameters and findings such as GFR, liver function tests, NT ProBNP, Albumin, INR, Electrolyte levels, edema, and Hb levels.

6. Are there any data available regarding the effects of using drugs that play an important role in the treatment of heart failure, such as SGLT2 inhibitors and Neprilysin inhibitors? If so, to what extent have they had an effect?

7. Has an analysis been conducted regarding the role of multimodal imaging techniques in predicting sudden cardiac arrest in patients with heart failure? Even if not, a discussion on this topic would be beneficial.

8. A significant shortcoming is that the samples include measurements at a single point in time and do not cover post-discharge follow-up or dynamic changes in clinical status.

9. There are also some challenges in using machine learning models in developing risk model. Deficiencies in clinical data and their recording, and the use of various data sources, can lead to decreased model performance. Clinicians who are knowledgeable about diseases, symptoms, and treatment methods may not be familiar with machine learning/artificial intelligence models, and those familiar with these models may not be familiar with their clinical interpretations. This can make it difficult to effectively apply the

obtained data to clinical and treatment processes. Therefore, approaches such as strengthening clinical integration, using explainable and hybrid models, and using larger and standardized data sources may be necessary. The authors' comments on this subject, considering existing studies and results, will enhance this article's contribution to the literature.

VERSION 1 - AUTHOR RESPONSE

Reviewer: 1
Dr. Shungo Hikoso, Nara Medical University
Comments to the Author:
This study addresses an important clinical question and applies a competing risk framework to predict cardiac arrest in patients with newly diagnosed heart failure. The use of cumulative incidence functions is appropriate and adds value compared with conventional approaches. However, several aspects related to outcome definition, prediction time horizon, variable transparency, and presentation currently limit the interpretability and clinical applicability of the results. I believe that further clarification and additional analyses are needed according to following comments..

Major comments

1. *The primary outcome is defined as cardiac arrest treated with CPR, while death without resuscitation is treated as a competing event. As acknowledged by the authors, death without CPR likely includes a substantial proportion of patients with DNACPR orders or those who transitioned to palliative care after discharge. In this setting, the competing event may largely reflect treatment decisions rather than true biological competing risk, which limits the interpretation of the model as predicting intrinsic cardiac arrest risk. To address this limitation more constructively, the authors may consider alternative or expanded outcome definitions. If registry data are available, inclusion of appropriate ICD or CRT-D therapies (e.g., appropriate shocks for ventricular tachyarrhythmias) as part of a composite outcome representing "aborted sudden cardiac death" could be explored. Such an approach may reduce dependence on CPR initiation and better capture clinically meaningful malignant arrhythmic events. At minimum, the feasibility and potential impact of this alternative outcome definition should be discussed, and sensitivity analyses using different outcome definitions could strengthen the clinical relevance of the findings.*

Thank you for this relevant and interesting comment. Unfortunately, we only have data on whether patients had received an ICD or CRT-D at hospital discharge. We chose to exclude this patient group along with patients discharged with palliative care to study the patient population that can potentially benefit from primary prevention against

cardiac arrest. We consider implantable defibrillators to be a form of primary prevention for cardiac arrest and that patients that have received an implantable defibrillator have already been identified as high-risk individuals. We aimed to identify additional patients at high-risk of cardiac arrest. Thus, including patients that have received implantable defibrillators in our machine learning model would be redundant.

2. *The current analysis evaluates outcomes over a long follow-up period of up to 10 years after discharge. For prediction of sudden cardiac arrest in heart failure, such a long time horizon may be strongly influenced by changes in clinical status, treatment, and goals of care over time, potentially diluting clinically relevant signals. To improve clinical interpretability, the authors may consider performing separate prediction analyses for clearly defined short-term horizons, such as 3, 6, and 12 months after discharge. Although the number of events would be smaller, these time frames may better reflect periods in which baseline clinical information is most relevant and competing risks related to treatment decisions are less dominant. Presenting model performance across these distinct time windows would help clarify whether predictive ability differs by follow-up duration.*

Thank you for this comment. We agree with the reviewer that the clinical data which are used as the features for the model may be less relevant for a cardiac arrest which has occurred beyond a year from hospital discharge due to changing health status. However, a significant limitation of our model is the limited number of cardiac arrests in the given observation time. By limiting the follow-up time as the reviewer suggests, we will likely reduce the number of cardiac arrest events even further and are unlikely to see an improvement in model performance.

One could imagine that DNACPR-orders and palliative treatment strategies are less likely to be implemented within one-year follow-up after hospital discharge. So, cardiac arrest events would be more likely to occur compared to the competing event death without resuscitation. However, when we have a closer look at figure 2, we find that the most frequent event is death without resuscitation and cardiac arrest remains to be a rare event within the first 5 years after hospital discharge. Thus, unfortunately limiting the follow-up time is not likely to change to model performance. As we discussed in the article, ideally, we would need updated information regarding the patients' health status over time, especially with regards to DNACPR-orders and transitions to palliative care.

With consideration to the reviewer's comments, we have highlighted an additional limitation of this study as follows:

Page 7, line 290-293: "**The model is constructed to predict cardiac arrest several years after the first hospitalization for heart failure. But the model includes predictors from this first hospitalization which may not be relevant for the**

prediction of a cardiac arrest event several years later. Additional updated data on the patient status from hospitalisations during the follow-up time would improve clinical relevance."

3. *The manuscript states that 82 predictors were included in the model, but Table 1 presents only a limited subset of baseline variables. It is currently unclear which variables were included in the model, at what time points they were measured (admission or discharge), and how much missing data was present for each predictor. Providing a complete list of predictors with basic information (definition, timing, and missingness), preferably as supplementary material, would substantially improve transparency and reproducibility.*

Thank you for this relevant suggestion. Data imputation resulted in 5% missingness in the data set. We have chosen to include a list of all included predictors as the reviewer suggests in supplementary material.

4. *Some predictors appear to represent closely related or repeated measurements (for example, admission and discharge values of similar laboratory parameters). While tree-based models can accommodate correlated variables, consideration of a more parsimonious predictor set, or sensitivity analyses using a reduced and clinically standard variable set, may improve interpretability and generalizability of the model.*

We thank the reviewer for this insightful comment. We agree that parsimonious models are generally preferable for interpretability and clinical applicability. However, in the present study, the two competing outcomes cardiac arrest and death without resuscitation, are biologically closely related. This similarity between outcomes makes discrimination challenging and places greater importance on capturing subtle differences in patient status. For this reason, we included both admission (reflecting initial severity) and discharge (reflecting treatment response and residual risk) measurements. Furthermore, as the reviewer comments, the random survival forest machine learning algorithm should remove redundant variables which do not add information to the decision trees prediction accuracy.
If the performance of a parsimonious model is equal to the original model with all included predictors, then this parsimonious model will have an increased interpretability and clinical applicability. However, this is only a relevant next step in the model development if the original model performed well. The present model did unfortunately not perform well due to the many limitations we have discussed. Thus, further analysis of a parsimonious model may not be relevant and is not likely to perform better than the model with all available predictors.

5. *The figures could be refined to better support interpretation of the results. In particular, Figure 2 would benefit from clearer visualization of the cumulative incidence*

functions and clarification of how time points were handled in estimation and plotting. Given the large sample size, it would be helpful to explain whether the stepwise appearance of the curves reflects the estimation method or time discretization. Minor improvements in figure presentation would enhance readability.

We thank the reviewer for this insightful comment. The cumulative incidence functions were estimated using the Aalen–Johansen estimator and are therefore presented as step functions reflecting observed event times. To improve interpretability while preserving the comparison between outcomes, we retained a single panel but added a zoomed inset to better visualize the less frequent cardiac arrest outcome. We have also tried to clarify the description of the methodology, interpretation and caption of the figure in the manuscript.

Page 4-5, line 156-159: "Cumulative incidence functions for cardiac arrest and death without resuscitation as first events **were estimated using the Aalen–Johansen estimator, accounting for the competing risk structure. The resulting cumulative incidence curves are presented as step functions reflecting changes at observed event times.**"

Page 6, line 211-215: **The cumulative incidence of cardiac arrest increased gradually over time and remained below 8% throughout follow-up, with a tendency to plateau after approximately 10 years. In contrast, the cumulative incidence of death without resuscitation increased substantially over time, exceeding 75% at 10 years (Figure 2). This marked difference in event frequency indicates that death without resuscitation was the dominant outcome in the cohort.**

Figure 2 caption: "Cumulative incidence for cardiac arrest and death in the study population of patients discharged after first hospitalization for heart failure. **Cumulative incidence functions were estimated using the Aalen–Johansen estimator and are displayed as step functions reflecting observed event times. Including a zoomed inset plot of the cumulative incidence of cardiac arrest.**"

6. *The overall narrative could be strengthened by more clearly aligning the study objective, analytical approach, and interpretation. Explicitly stating whether the primary aim is biological risk prediction of sudden cardiac arrest or prediction of CPR-treated cardiac arrest would help readers better understand the scope and limitations of the study.*

Thank you for this relevant comment. Our primary aim was to predict the biological event cardiac arrest. But in truth our model is predicting cardiac arrest which received cardiopulmonary resuscitation. The latter outcome would not be relevant for the

downstream purpose of enabling cardiac arrest prevention. We attempted to clarify this distinction in the discussion of our results.

Page 7, Line 278-284: “Ideally, patients who transitioned to palliative care or received DNACPR-orders should be excluded from observation from then on. In such a case, a composite endpoint of cardiac arrest in SRCR and death without resuscitation would include all cardiac arrests eligible for CPR and preventative measures, regardless of whether the patient actually received CPR. **Such an outcome would represent the biological event of cardiac arrest which we had primarily intended to predict. But the current model is in fact predicting cardiac arrests treated with CPR, a non-biological event.**”

Reviewer: 2
Dr. Tahir Yagdi, Ege Universitesi
Comments to the Author:
I carefully read the article titled “Prediction of cardiac arrest in patients with heart failure – development of a machine learning model" sent to me for review. My comments, criticisms, and suggestions are listed below:

1. *According to the literature, third-degree AV block and left bundle branch block are considered significant predictors of sudden cardiac arrest. Do the authors have any findings regarding this? A discussion on this topic would be appropriate. Similarly, the impact of the presence of paroxysmal or long-term atrial fibrillation should be evaluated.*

Thank you for this comment. The Swedish heart failure registry did not register specific data on the presence of third-degree AV block. However, we did have data on left bundle branch block as well as the presence of atrial fibrillation. These variables were included as potential predictors for the model, yet it did not seem to have enough variable importance for the model prediction as according to figure 4. But we have chosen to include the variables which we have available data in the baseline table (table 1).

2. *Possible mechanisms of sudden cardiac arrest include left ventricular hypertrophy, neurohormonal activation, myocardial ischemia, and cardiac fibrosis. Have these findings been analyzed? I would appreciate comments on this.*

We thank the reviewer for this insightful comment. An additional analysis of cardiac arrest subdivided into the known aetiology would be interesting. However, we do not have the data to do this analysis. This could be a relevant research question for future

investigations if the biological event cardiac arrest can be better defined as an outcome to study.

3. *Can any comments be made regarding the impact of heart transplantation and mechanical circulatory support system implantation, which are the gold standard surgical treatment methods in the surgical treatment of end-stage heart failure, on the outcomes?*

We are grateful for this interesting comment. A limitation of our study design is that we are limited to the Swedish heart failure registry's data collection and variable selection. There is no available data on heart transplantation and mechanical circulatory support system implantation in data which we had extracted from the registry. As such we are unable to comment on the correlation between this surgical treatment and cardiac arrest outcome.

4. *Have the impacts of the number and frequency of cardiac-related hospital admissions been evaluated? Have the changes in treatment during these admissions been assessed? Have any potential changes affected outcomes? I suggest this for discussion.*

Thank you for this comment. Unfortunately, we do not have data on the transitions and changes in patient status over time after the first hospitalization. This is a limitation of our model which we have decided to highlight in the discussion.

Page 7, line 290-293: "**The model is constructed to predict cardiac arrest several years after the first hospitalization for heart failure. But the model includes predictors from this first hospitalization which may not be relevant for the prediction of a cardiac arrest event several years later. Additional updated data on the patient status from hospitalisations during the follow-up time would improve clinical relevance**."

5. *It would be beneficial to discuss, in light of the literature, the impact of changes over the years on the results of parameters and findings such as GFR, liver function tests, NT ProBNP, Albumin, INR, Electrolyte levels, edema, and Hb levels.*

We agree with the reviewer's comments that this would be a relevant aspect to discuss and investigate. However, we do not have data on the changes in laboratory parameters beyond the first hospitalization for heart failure. Future investigations should certainly consider a more extensive data collection to include changes over the years as the reviewer suggests.

Page 7, line 290-293: "**The model is constructed to predict cardiac arrest several years after the first hospitalization for heart failure. But the model includes predictors from this first hospitalization which may not be relevant for the prediction of a cardiac arrest event several years later. Additional updated data on the patient status from hospitalisations during the follow-up time would improve clinical relevance**."

6. *Are there any data available regarding the effects of using drugs that play an important role in the treatment of heart failure, such as SGLT2 inhibitors and Neprilysin inhibitors? If so, to what extent have they had an effect?*

Thank you for this relevant comment. We had included data from the Swedish heart failure registry which included data on which medications relevant for heart failure treatment had been prescribed upon hospital discharge or given during the hospital stay. This included Angiotensin-receptor-Neprilysin inhibitors, ACE-inhibitors, anticoagulants, antiplatelet medication, beta-blocker, Digoxin, ferric carboxymaltose, inotrope, loop-nitrate, loop-diuretic, Mineralocorticoid receptor antagonists, SGLT2-inhibitors, Ivabradin and HMG-CoA reductase inhibitors (statins). From what we can see in figure 4, these medications do not seem to be important for this current model prediction of cardiac arrest.

We have provided an extensive list of all the included predictors in the supplementary materials.

7. *Has an analysis been conducted regarding the role of multimodal imaging techniques in predicting sudden cardiac arrest in patients with heart failure? Even if not, a discussion on this topic would be beneficial.*

Thank you for this interesting perspective which you suggest in your comment. We have not performed any such analysis because we unfortunately do not have any detailed data from multimodal imaging techniques. The Swedish heart failure registry collects categorical data on Chest x-ray results indicating heart enlargement and/or pulmonary statis or none of the aforementioned. We also have data on the method used to estimate the left ventricular ejection fraction, the numeric value of LVEF and the categorical variable of LVEF (Severe, <30%, Moderate, 30-39%, Mild, 40-49%, Normal, ≥50%). The current model did not find these variables to be of high importance for the model prediction, as can be seen in figure 4.

8. A significant shortcoming is that the samples include measurements at a single point in time and do not cover post-discharge follow-up or dynamic changes in clinical status.

We agree with the reviewer’s comments. Unfortunately, we are limited by the data that is available to us in the Swedish heart failure registry. We have taken this limitation into account and highlighted it in the discussion.

Page 7, line 290-293: “**The model is constructed to predict cardiac arrest several years after the first hospitalization for heart failure. But the model includes predictors from this first hospitalization which may not be relevant for the prediction of a cardiac arrest event several years later. Additional updated data on the patient status from hospitalisations during the follow-up time would improve clinical relevance**.”

9. *There are also some challenges in using machine learning models in developing risk model. Deficiencies in clinical data and their recording, and the use of various data sources, can lead to decreased model performance. Clinicians who are knowledgeable about diseases, symptoms, and treatment methods may not be familiar with machine learning/artificial intelligence models, and those familiar with these models may not be familiar with their clinical interpretations. This can make it difficult to effectively apply the obtained data to clinical and treatment processes. Therefore, approaches such as strengthening clinical integration, using explainable and hybrid models, and using larger and standardized data sources may be necessary. The authors' comments on this subject, considering existing studies and results, will enhance this article's contribution to the literature.*

Thank you for this insightful comment. We agree with the reviewer’s comments that to bridge the gap between machine learning/artificial intelligence models and clinical practice is difficult. The present study aims to develop a machine learning model based on registry data. Such a model can be further evaluated and improved on clinical data before potential clinical implementation. The present study is therefore intended to be a starting point. However, the current model performs poorly in discriminating between cardiac arrest and death without resuscitation due to data limitations. These data limitations have resulted in an outcome cardiac arrest defined as cardiac arrest which was treated with CPR rather than the biological event cardiac arrest. The present study highlights which necessary data we need for developing clinically meaningful cardiac arrest prediction models, which remains to be a highly relevant future endeavour.

Page 8, line 317-322: “**Currently published studies on machine learning models developed for cardiac arrest prediction are based on dynamic time-sensitive physiological data such as vital signs, ECG, etc. But our approach uses static, baseline data and known clinical risk factors to develop a machine learning prediction model. If such a model performed well and superior to existing risk models, it would be clinically useful for risk stratification and improved resource allocation**.”

VERSION 2 - REVIEW

**Reviewer**	**1**
**Name**	**Hikoso, Shungo**
**Affiliation**	**Nara Medical University, Department of Cardiology**
**Date**	**06-May-2026**
**COI**	

The authors added several descriptions and data in response to this reviewer's comments. Although there still remains several limitations, the authors improved the manuscript substantially. This reviewer has no further comments.

**Reviewer**	**2**
**Name**	**Yagdi, Tahir**
**Affiliation**	**Ege Universitesi**
**Date**	**15-Apr-2026**
**COI**	

I thank the authors for their thoughtful responses and explanations to my comments, criticisms, and suggestions.

VERSION 2 - AUTHOR RESPONSE

Reviewer: 1
Dr. Shungo Hikoso, Nara Medical University
Comments to the Author:
The authors added several descriptions and data in response to this reviewer's comments. Although there still remains several limitations, the authors improved the manuscript substantially. This reviewer has no further comments.

Reviewer: 2
Dr. Tahir Yagdi, Ege Universitesi
Comments to the Author:
I thank the authors for their thoughtful responses and explanations to my comments, criticisms, and suggestions.
